# Universal health coverage in the context of migration and displacement: a cosmopolitan perspective

**DOI:** 10.1016/S2468-2667(25)00117-3

**Published:** 2025-06-16

**Authors:** Santino Severoni, Claudia Marotta, Josephine Borghi

**Affiliations:** a Department of Health and Migration, Health and Migration Programme, Division of Universal Health Coverage and Healthier Populations, WHO, Geneva, Switzerland; b Social Cohesion, Health, and Wellbeing Research Group, International Institute for Applied Systems Analysis, Laxenburg, Austria; c Department of Global Health and Development, London School of Hygiene & Tropical Medicine, London, UK

## Abstract

Migration and displacement are reshaping societies and economies with profound implications for health equity and universal health coverage (UHC). In this Viewpoint, we review the unique health challenges faced by migrants and displaced people, as well as the limitations of current UHC policies and financing arrangements. We propose a cosmopolitan approach to UHC, grounded in global solidarity and structured around four pillars: supranational financing, integrated cross-border care, harmonised legal frameworks, and long-term investment in inclusive health systems. We also explore what this approach could mean practically for regional or global financing mechanisms and sources of funding, including progressive contributions and the integration of health into climate finance. Achieving equitable and effective UHC in a world shaped by mobility and crisis requires global thinking and collective action. We call for a reimagining of UHC via a cosmopolitan approach, which offers a pathway to reframe health and wellbeing as a shared right and responsibility, transcending national borders.

## Introduction

Migration and displacement are shaping societies and economies worldwide. Although often framed through numbers, migration is far more than a demographic trend, reflecting deeply intertwined factors: conflict, inequality, political instability, environmental changes, and aspirations for a better life.

To date, more than 1 billion people—one in eight people globally—are on the move or displaced, driven by a range of factors, such as opportunity, crisis, or inequality. This figure includes approximately 304 million international migrants, who live outside their country of birth,^1^ and 763 million internal migrants, who have relocated within national borders,^2^ often facing similar vulnerabilities and service gaps. Within these broader categories are 122·6 million people who have been forcibly displaced, uprooted by war, persecution, and violence.^3^ This group includes 37·8 million refugees and others in need of international protection.^3^ These categories are distinct yet overlapping; therefore, the number of people should not be summed directly. Nonetheless, the data underscore the immense and multifaceted scale of global human mobility.

Migration is increasingly shaped by global trends, such as climate change, which is displacing millions of people through rising sea levels, extreme weather events, and resource scarcity. These environmental pressures—compounded by demographic shifts and persistent inequalities—are projected to cause continued and, in some regions, exponential increases in displacement, particularly across Africa and Asia.^4^ Forced migration is increasingly protracted, due to the enduring nature of many conflicts,^5^ and climate stressors pushing communities beyond adaptation limits, making it difficult to return home. These evolving migration patterns have profound implications for global health, demanding urgent and coordinated policy responses.

In this Viewpoint, we argue that current universal health coverage (UHC) policies and financing arrangements might fall short in addressing the unique health needs of migrants and displaced populations. We call for a reimagining of UHC—one that transcends national borders and reflects our shared responsibility for human wellbeing in an increasingly interconnected and rapidly changing world.

## Gaps in UHC: migration and displacement

Migrants and refugees can face unique health challenges across the migration cycle, shaped by the conditions of departure, the journey itself, and post-arrival experiences. Many people flee war, persecution, or natural disasters, which increases risk to physical and mental health and limits access to health care in their home countries. The WHO world report on the health of refugees and migrants^6^ emphasises that migration and displacement are health determinants. Refugees and migrants are exposed to a complex set of health risks across the migration cycle, shaped by individual, environmental, and socioeconomic factors.^6^

Barriers to diagnosis and treatment increase vulnerability to antimicrobial resistance and delayed HIV care.^6^ Mental health conditions—including depression, anxiety, and post-traumatic stress disorder—are prevalent, especially among those people exposed to trauma.^6^ Occupational health risks are also high, as many migrants work in hazardous, informal, or poorly regulated sectors.^6^ Additionally, non-communicable diseases, such as cancer, diabetes, and hypertension, are frequently undiagnosed, untreated, or poorly managed compared with host populations.^6^

The journey of migration or displacement can be perilous, marked by malnutrition, exposure to disease, and physical or emotional trauma. Upon arrival in host countries, migrants and refugees often encounter systemic barriers to health care—including legal restrictions, financial constraints, language barriers, and discrimination.^6^ Refugees frequently spend extended periods in camps or informal settlements where access to essential services, such as health care, clean water, and sanitation, remains scarce.

The COVID-19 pandemic starkly highlighted these vulnerabilities. Migrants had increased health risks due to their living and working conditions,^7^ as well as barriers to early vaccine access, as few countries prioritised migrants and refugees.^8^ Even when vaccines became widely available, uptake remained lower among migrants and refugees than host populations due to barriers such as lack of trust, language and cultural differences, and limited access to health-care services.^9^

UHC should be central to migration-related and refugee-related health policies.^10^, ^11^ Ensuring migrants and refugees have access to affordable and quality health care not only upholds human rights, but also strengthens public health. The health of these populations is inextricably linked to the health of host communities. Investing in migrant and refugee health care is a pragmatic necessity.

However, migration is too often framed as a threat, with governments prioritising border security over humanitarian considerations. Health systems are increasingly being tasked with enforcing national borders, such as by verifying a person's legal status before providing care—a practice known as bordering, with the aim to reduce costs for host countries. This practice contradicts the ethos of UHC, which is based on providing care according to need, not nationality or legal status.^12^ This enforcement has led to restrictive policies undermining migrant and refugee access to health care, with the benefits offered also being substantially restricted in some settings, especially for undocumented migrants. Undocumented migrants and refugees often avoid seeking medical care due to fear of deportation, therefore compromising their individual health and creating public health blind-spots that are important to address from a global health security perspective.

Although current international frameworks—such as the Global Compact for Safe, Orderly, and Regular Migration or the Global Compact on Refugees—recognise health as a crucial aspect of migration and call for equitable burden-sharing and responsibility-sharing, they fall short in proposing an operational model for UHC.

As a result, comprehensive health financing for migrants and refugees remains a persistent challenge. In relation to health financing, international burden-sharing (primarily through official development assistance [ODA]), has proven insufficient to address the increasing number of displaced populations and their health-care needs. In the case of refugees, ODA for health is estimated to be 5% of all refugee ODA,^13^ or US$23 per person per year.^14^ Furthermore, ODA mechanisms often prioritise immediate relief over long-term health system strengthening.^14^ Allocations of ODA for migrant health are inequitable, as some countries hosting large numbers of migrants, such as Iran and Pakistan, receive proportionally less aid than what is needed. Furthermore, over two-thirds of ODA contributions come from just five donors.^13^ The announcements of aid cuts from three of these donors (USA, UK, and EU) will, therefore, substantially reduce ODA for migrant and refugee health going forward. There is substantial variation in the health-care benefits funded for migrants and refugees, with some countries offering care similar to that of host populations, and other countries restricting coverage to emergency or lifesaving care. Although global funding initiatives for more equitable burden-sharing have been established, such as the Global Concessional Financing Facility, allocations are determined by project submissions, which, to date, often do not include health.

With reductions in UN High Commissioner for Refugees funding,^15^ and moves towards integration of migrant and refugee health financing mechanisms into national systems,^16^ host countries have an increasingly important role in funding health care for migrants and refugees. WHO^17^ highlight ongoing efforts and initiatives in health-care delivery for migrants and refugees across various regions, including resource-constrained settings. For example, Uganda provides refugees with entitlement to the same health-care access as nationals,^18^ and is transferring ownership of non-governmental organisation facilities providing services to refugees to the government, with funding from district budgets. Iran enables registered refugees to access the national health insurance scheme and offers free primary health care to undocumented migrants.^19^ Thailand offers health insurance to migrants, including undocumented migrants.^20^ As a result, many countries have shown improved health indicators, including expanded vaccine coverage, reduced maternal mortality rates, and strengthened public health infrastructures for refugees and migrants. These examples underscore the potential for collaborative efforts to yield health benefits for all, including migrants and refugees.

However, these arrangements are not without challenges. Undocumented migrants (eg, in Iran)^19^ and refugees (eg, in Thailand)^21^ are being excluded from health insurance coverage, and premium affordability and other barriers are limiting enrolment among those eligible for cover.

Furthermore, the increasing reliance on host-country funding creates disproportionate responsibilities for financing migrant and refugee health care, which, in the case of low-income and middle-income countries, can risk undermining UHC progress. A shift towards equitable financing models that mobilise adequate resources to ensure financial protection for migrants, while also promoting long-term health system strengthening, is essential.

## Towards a new UHC paradigm

To address the outlined challenges, a UHC framework based on cosmopolitan principles is needed, whereby the UHC perspective shifts from a national to a global scale.^22^ A cosmopolitan perspective views individuals as part of a shared global community, emphasising universal moral obligations and collective responsibility that transcends national borders. A UHC approach based on cosmopolitan principles should prioritise equity in health-care contributions and allocations to promote UHC globally, regardless of migratory status (panel).

Panel **Cosmopolitan approach to universal health coverage**

1 Global solidarity mechanisms: countries contribute based on ability to pay with resources pooled at the supranational level to maximise efficiency and redistribution. Resources are allocated to host countries based on need, and covering a universal essential benefit package for migrant populations.
2 Integrated health networks: cross-border health-care cooperation to ensure continuity of care for mobile and displaced populations.
3 Universal access policies: harmonised legal frameworks guaranteeing health coverage for migrants and refugees across jurisdictions.
4 Long-term investments: resources allocated to preventive care, mental health services, and infrastructure addressing immediate and structural health needs.

As most migration occurs within regions, a regional UHC approach could be adopted. Existing models, such as the EU's cross-border health-care system, offer useful precedents. In the EU, European Health Insurance Cards (EHICs) provide access to care across member states, with hosting countries reimbursed by countries of origin. Similar arrangements could be adapted for other regional or economic organisations (such as the Association of Southeast Asian Nations, the North American Free Trade Agreement, or the African Union) or cooperative platforms focused on migration (eg, Intergovernmental Authority on Development, the Comprehensive Regional Protection and Solutions Framework, and the Support Platform for the Solutions Strategy for Afghan Refugees).

However, there are challenges with such an approach. In the EU, uptake of EHICs remain uneven, particularly in less wealthy countries. Furthermore, differences in health-care costs across countries create a financial burden for less wealthy countries with large migrant populations.^23^ These disparities reduce the applicability of such models in low-income regions, unless a regional risk pool is established to offset cost differentials. If migration remains concentrated within low-income regions, the financial burden of migrant health care is disproportionately placed on the countries least able to afford it, therefore undermining the principles of UHC.

These challenges point to the need for a global health financing pool. This pool could be funded by compulsory and progressive (income-based) country contributions, alongside funding from innovative financing mechanisms; for example, earmarked global taxes on multinational corporations. As climate-induced migration increases, climate finance—specifically, from loss and damage or adaptation funds—could cofinance migrant health care, providing an additional source of funding. Global agreement would be needed on a minimum level of essential services guaranteed to migrants everywhere, with funding allocated based on need and health-care use via existing financing mechanisms to avoid inefficiencies introduced by parallel funding systems.

Lastly, health-care need is both a driver and consequence of migration; as such, measuring the health dimensions of migration more effectively will be essential.

## Conclusion

Migration and forced displacement are not crises to be solved, but realities to be managed. Although a cosmopolitan approach to UHC is undoubtedly challenging amid rising nationalism, aid cuts, and the erosion of multilateral cooperation, the health of migrants and refugees offers a powerful entry point to advocate for renewed global solidarity and sharing of responsibilities. The health of an individual is inherently connected to the health of all. In a world increasingly shaped by climate change, conflict, and economic instability, everyone is, potentially, a migrant—responsibility for, and conceptions of, UHC must therefore extend beyond national borders, to meet transnational challenges and sustain progress towards UHC globally. In this Viewpoint, we have proposed a new vision for UHC grounded in cosmopolitan principles, and have begun to outline potential pathways for implementation. To ensure equitable and effective health systems and UHC, we should begin to think and act globally to build a world where health care is a right for all, regardless of borders.

### Search strategy and selection criteria

We searched PubMed and Google Scholar databases for studies published in English between Jan 1, 2018, and Dec 31, 2024, complemented by manual reviews of reference lists from key papers and reports. This timeframe was selected to capture the most recent evidence, including key developments related to the COVID-19 pandemic, displacement linked to climate change, and evolving universal health coverage (UHC) frameworks. Additional searches targeted grey literature, policy briefs, and reports from leading institutions and platforms, such as WHO, UN High Commissioner for Refugees, International Organization for Migration, and the UHC 2030 Platform. We used the following search terms in various combinations: “universal health coverage” OR “UHC” AND (“migrants” OR “refugees” OR “asylum seekers” OR “displaced persons”) AND (“health access” OR “health financing” OR “health system”). Inclusion criteria encompassed studies and reports that addressed the intersection of migration or displacement and UHC-related health system design, access to services, or financing models. We selected additional articles and policy documents, especially where empirical gaps existed in the peer-reviewed literature.

### Contributors

## Declaration of interests

CM and SS are employed at the Department of Health and Migration, WHO. The authors alone are responsible for the views expressed in this Viewpoint, which do not necessarily represent the decisions, policy, or views of WHO.
